# Exploiting B7-H3: Molecular Insights and Immunotherapeutic Strategies for Osteosarcoma

**DOI:** 10.3390/bioengineering12121344

**Published:** 2025-12-10

**Authors:** Yuhang Xie, Hongru Wang, Fanwei Zeng, Yuan Zhang, Jiaye Huang, Chenglong Chen, Shidong Wang

**Affiliations:** 1 Peking University International Hospital, Peking University Health Science Center, Beijing 102206, China; 2 Department of Neurology, Liaocheng People’s Hospital, Liaocheng 252000, China; 3 Musculoskeletal Tumor Center, Peking University People’s Hospital, Beijing 100044, China; 4 Peking University People’s Hospital, Peking University Health Science Center, Beijing 100044, China; 5 Department of Orthopaedic Oncology Surgery, Beijing Jishuitan Hospital, Capital Medical University, Beijing 100035, China

**Keywords:** B7-H3 (CD276), Osteosarcoma, immunotherapy, chimeric antigen receptor T-cell therapy, antibody-drug conjugates

## Abstract

Osteosarcoma (OS) remains the most common primary malignant bone tumor in adolescents, with conventional treatments yielding only modest improvements in long-term survival. Immunotherapy has emerged as a promising strategy to overcome these limitations. B7-H3 (CD276) stands apart from other potential targets due to its high expression in tumors cells, as well as its strong association with tumor aggressiveness and poor prognosis. This review provides a comprehensive overview of B7-H3, covering its molecular structure, regulatory mechanisms, biological functions, and expression patterns in tumor tissues. We emphasize the dual roles of B7-H3—both immunoregulatory and non-immunoregulatory—in shaping the tumor microenvironment (TME) and facilitating immune evasion. Building on these insights, we summarize current immunotherapeutic strategies targeting B7-H3 in OS, including monoclonal antibodies (mAbs), chimeric antigen receptor T cells (CAR-T), antibody-drug conjugates (ADCs), and bispecific antibodies (bsAbs). These four strategies have their own advantages and deficiencies. Excitingly, rapid advances in nanoparticle-based systems offer promising solutions to overcome the limitations, especially to develop more effective drug delivery systems and to reshape the TME by targeting immune cells. Despite promising progress, significant challenges remain. These include the absence of an identified B7-H3 receptor, the immunosuppressive and heterogeneous nature of the OS TME, and the need for improved targeting specificity and safety. Addressing these challenges through optimization of delivery systems, combination strategies, and the integration of nanotechnology may unlock the full potential of B7-H3-based immunotherapy in the treatment of OS.

## 1. Introduction

Osteosarcoma (OS) is a malignant bone tumor that originates from mesenchymal cells, with an incidence rate of approximately 2–5 per million globally [1]. It is the most prevalent primary malignant bone tumor in children and adolescents [2,3]. Around 75% of OS patients are under the age of 25, with a median age at diagnosis of approximately 20 years, which correlates with rapid skeletal growth and hormonal changes during puberty [4,5]. Epidemiological studies reveal a bimodal age distribution, with a primary peak around 24 years of age and a secondary peak in older adults around 70 years [5,6]. Unlike primary OS in adolescents, OS in older adults is often secondary to conditions such as Paget’s disease or prior radiation exposure [5]. In addition, genetic factors, chronic radiation exposure, and contact with certain chemicals (e.g., beryllium, radium, chromium) have been identified as risk factors for the development of OS [7].

OS predominantly affects the metaphysis of long bones and has a high propensity for pulmonary metastasis. Since the 1980s, surgery combined with chemotherapy has become the standard treatment for OS [6]. Over the years, ongoing research has sought to refine and advance chemotherapeutic approaches, culminating in the current standard treatment, which includes neoadjuvant chemotherapy followed by surgical resection. Since the establishment of the standard chemotherapy regimen, the prognosis for patients with localized OS has improved, with a five-year survival rate of approximately 60–70%. However, patients with metastatic disease—most commonly to the lungs—or recurrence OS have a significantly poorer prognosis, with five-year survival rates of only 20–30% [3,8,9,10]. In recent years, progress in improving the five-year survival rate for OS patients has plateaued, and the management of this disease remains a significant clinical challenge.

In recent years, there has been an increased emphasis on exploring novel therapeutic approaches to address the challenges in OS treatment. This includes intensified research into targeted therapy, immunotherapy, stem cell therapy, and photodynamic therapy, all of which have gained significant momentum. These approaches have shown promising results in various solid tumors, offering new hope for the treatment of OS [3]. With growing understanding of the OS tumor microenvironment (TME), which is characterized by various infiltrating cells and dysregulated signaling pathways, immune intervention is now supported by a stronger biological foundation. As a result, immunotherapy emerges as a particularly promising and advanced avenue in drug development.

The concept of tumor immunotherapy dates back to the late 19th century, when the American physician William Coley observed that some cancer patients survived longer after postoperative bacterial infections. This observation led him to hypothesize that the immune activation could inhibit tumor growth [11]. Contemporary immunotherapy strategies are based on the TME of OS, which plays a critical role in immune evasion by OS cells [12]. Compared to conventional chemotherapy, immunotherapy has the potential to overcome drug resistance and immune evasion, reduce adverse effects, and improve treatment outcomes through more precise targeting and activation of antitumor immune responses [3,13]. However, the high heterogeneity and immunosuppressive nature of the OS TME remain significant barriers to the clinical success of immunotherapy [3].

Current immunotherapy strategies encompass a variety of modalities, including immune checkpoint inhibitors (ICIs), tumor vaccines, adoptive cell therapy, oncolytic virus therapy, and immunomodulators [3,10,13]. However, not all of these strategies have been widely adopted in clinical practice for OS. In recent years, research on immune checkpoints has primarily focused on PD-1, PD-L1, and CTLA-4, which are the main targets in clinical applications. While agents targeting these checkpoints have shown efficacy in solid tumors, concerns regarding endocrine disorders, skin irritation, gastrointestinal reactions, and joint pain [14] have been raised regarding their use in OS. As a result, the exploration of novel immune checkpoints has become a central area of research. Numerous studies have demonstrated that B7-H3 is overexpressed in cases of OS and other solid tumors and is closely associated with tumor metastasis and poor prognosis. These findings highlight the unique potential of B7-H3 as a target for immunotherapy.

## 2. Overview of B7-H3

### 2.1. Molecular Structure and Regulation of Expression

**Figure 1:**
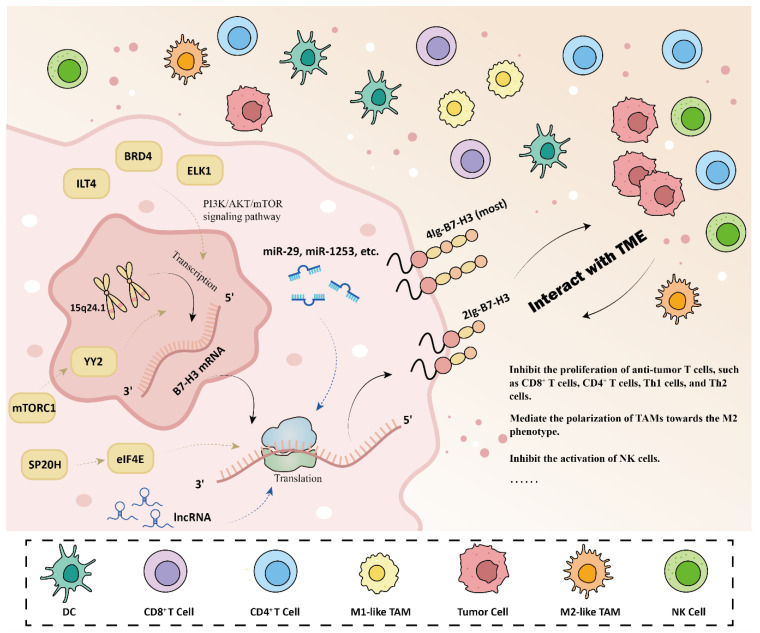
The expression and function of B7-H3. The gene is located on 15q24.1, and B7-H3 mRNA is widely present in most normal tissues, while B7-H3 protein is highly expressed only under pathological conditions. The regulatory mechanisms of B7-H3 expression include (1) BRD4, ILT-4, and ELK1, which regulate B7-H3 expression through the PI3K/AKT/mTOR signaling pathway. (2) Overactivation of mTORC1 in tumor cells has been shown to increase B7-H3 expression through a direct phosphorylation of the transcription factor YY2. (3) mRNAs, like miR-29 and miR-1253, function as regulatory agents. (4) lncRNAs regulate the expression of B7-H3 in macrophages. (5) SP20H regulates B7-H3 negatively, while eIF4E regulates B7-H3 positively. The activation of the P38 MAPK-eIF4E signaling pathway represents a key step in the process of transcriptional initiation and protein expression of B7-H3 in tumor cells. The function of B7-H3 includes immune and non-immune functions. The interaction between B7-H3 and TME is complicated.

Figure 1 summarizes the expression and function of B7-H3. Also known as CD276, B7-H3 belongs to the B7 protein family and the B7/CD28 superfamily. It is a type I transmembrane glycoprotein, which is highly conserved across species. First cloned from a human dendritic cell cDNA library in 2001, B7-H3 shares 20–27% amino acid sequence homology with other members of the B7 co-stimulatory family [15]. Structurally, B7-H3 features a typical immunoglobulin-like structure, consisting of an extracellular domain and an intracellular domain. The extracellular domain contains both immunoglobulin constant-like (IgC) and immunoglobulin variable-like (IgV) domains. Based on variations in this region, two distinct isoforms of B7-H3 have been identified: the 4Ig-B7-H3 variant, which contains two identical pairs of IgC and IgV domains, and the 2Ig-B7-H3 variant, which contains one set of each. The 4Ig-B7-H3 isoform is the predominant form found in humans [16,17,18]. Although some studies suggest that the two isoforms may have unelucidated functional differences [19], the majority of research on B7-H3 expression and function does not differentiate between them [20].

It is noteworthy that classical immune checkpoints, such as CTLA-4 and PD-L1, are also members of the B7 protein family. This family consists of integral membrane proteins, primarily expressed on activated antigen-presenting cells (APCs), which modulate immune responses through interactions with corresponding receptors on lymphocytes [18]. Functionally, the B7 family—the focus of this review—can be divided into two categories: co-stimulatory proteins (e.g., B7-1, B7-2, B7-H2) and co-inhibitory proteins (e.g., B7-H4, PD-L1, CTLA-4). These proteins play a critical role in regulating immune balance and modulating MHC-TCR signaling between APCs and T cells [21]. Recent advances in tumor immunology have established tumor-infiltrating T lymphocytes as the primary antitumor effector cells within the TME. However, their activity can be suppressed by co-inhibitory signals derived from B7 family proteins expressed on their surface, thereby promoting immune evasion by the tumor cells. This mechanism underscores the potential of targeting the B7 family in novel immunotherapeutic strategies.

The gene encoding the IgC and IgV domains of the B7-H3 protein is located on chromosome 15q24.1 [22]. While B7-H3 mRNA is present in both immune and non-immune normal tissues, its protein expression remains low under normal physiological conditions [17]. This discrepancy highlights the significance of post-transcriptional regulatory mechanisms in controlling B7-H3 expression. Multiple mechanisms regulate B7-H3 expression, with distinct regulatory patterns observed in different tumor cells. MicroRNAs (miRNAs) are small non-coding RNA molecules that act as key regulators of gene expression. In various cell types, a negative correlation has been observed between miR-29, miR-1253, and B7-H3 expression levels [21]. Studies have shown that miR-29 reduces the expression of B7-H3 by binding to the 3′ untranslated region (UTR) of B7-H3 mRNA [16,17,18,23]. The findings of these studies suggest that post-transcriptional regulation plays a pivotal role in the expression of B7-H3. In addition to miR-29, other miRNAs, such as miR-199a, miR-128, and miR-187, have been found to directly or indirectly bind to the B7-H3 3′ UTR mRNA, thereby modulating its expression levels. Long non-coding RNAs (lncRNAs) further contribute to this regulatory network. For instance, in macrophages, lncRNA TUG1 inhibits miR-29, resulting in the upregulation of B7-H3 expression. Similarly, lncRNA NEAT1 enhances the expression of B7-H3 and promotes the polarization of macrophages towards the M2 phenotype by the sponging of miR-214 [21]. At the signaling level, B7-H3 expression is regulated by BRD4, ILT-4, and ELK1 through the PI3K/AKT/mTOR pathway, affecting both transcription and epigenetic regulation [18,21]. These results have been confirmed in cell lines of lung cancer, pancreatic ductal adenocarcinoma, renal cell carcinoma, colorectal cancer, and cervical cancer [18]. In tumor cells, overactivation of mTORC1 enhances B7-H3 expression by directly phosphorylating the transcription factor YY2 [21]. Moreover, studies in HEK293T, SK-OV-3, and HeLa cell lines have demonstrated that SUPT20H (SP20H) exerts a negative regulatory effect on B7-H3, while eIF4E has been shown to positively regulate B7-H3. The activation of the P38 MAPK-eIF4E pathway is a key step in the process of transcriptional initiation and protein expression of B7-H3 in tumor cells [24]. Overall, B7-H3 expression is influenced by a complex network of post-transcriptional modifications and translational regulatory mechanisms.

### 2.2. The Function of B7-H3

**Figure 2:**
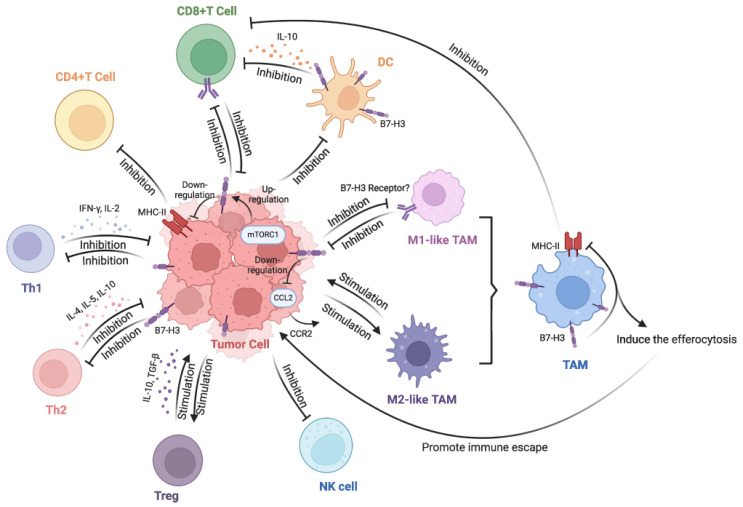
The interaction of tumor cells expressed B7-H3 with immune cells. Reproduced with permission for Ref. [21]. Copyright © 2025, The Author(s).

Under physiological conditions, the B7-H3 protein is expressed at low levels, but it is widely present in both lymphoid and non-lymphoid tissues. It is inducibly expressed on immune cells. including T cells, natural killer (NK) cells, and APCs such as dendritic cells (DCs) and macrophages, where it exerts immunosuppressive functions [25]. In contrast, B7-H3 is typically highly expressed on tumor cells, where it not only promotes tumor cell immune evasion by inhibiting immune responses but also mediates tumor metastasis, drug resistance, and angiogenesis through non-immune mechanisms [15,16,18,26]. Within the TME, B7-H3 has been shown to suppress T cell–mediated immune responses, promote the polarization of tumor-associated macrophages (TAMs) towards the M2 phenotype, and also inhibit the functions of other immune cells (Figure 2) [21]. Both immunoregulatory and non-immunoregulatory functions have been summarized in this part. However, it is noteworthy that which pathway is dominant in OS is not fully characterized. The discussion that follows can then be positioned as inferences based on general cancer biology and initial findings.

#### 2.2.1. Immunoregulatory Function

Human B7-H3, both in the 2Ig and 4Ig isoforms, has been shown to inhibit T cell proliferation and to downregulate the expression of certain cytokines. It suppresses the activation and expansion of CD4^+^ and CD8^+^ T cells by negatively regulating key transcription factors such as NFAT, AP-1, and NF-κB, while also reducing IL-2 secretion [18]. Conversely, some studies suggest that B7-H3 may exert a positive regulatory effect on Th2 and Th17 cells, enhancing the secretion of cytokines including IL-4, IL-10, and IL-17. B7-H3 expression has been shown to promote the formation of an immunosuppressive TME by increasing the production of IL-10 and TGF-β1, while inhibiting the activity of various immune cells, including CD4^+^ T cells, CD8^+^ T cells, and DCs [18]. Research has also demonstrated that elevated levels of B7-H3 in tumor samples correlate with increased Treg cell populations, suggesting that B7-H3 has an inhibitory effect on Treg cell activity [2]. Additionally, B7-H3 has been linked to the differentiation of TAMs, with the potential to induce their polarization and immunosuppression via the CCL2-CCR2-M2 macrophage axis [22]. However, the specific receptor for B7-H3 remains unidentified. Nevertheless, studies have proposed that B7-H3 may interact with a receptor expressed on activated CD4^+^ T cells and CD8^+^ T cells, which could be recognized by B7-H3 expressed on APCs or tumor cells [18]. Although B7-H3 is widely recognized as immunosuppressive, its function may depend on the receptor context. B7-H3 was originally described as a potent costimulatory molecule and inducer of IFN-γ in human T cells [27]. It is possible that different receptors mediate distinct immune functions [28].

#### 2.2.2. Non-Immunoregulatory Function

Beyond its immunoregulatory roles, B7-H3 contributes to tumor progression through multiple non-immune mechanisms, including the promotion of tumor cell proliferation and metastasis, stimulation of angiogenesis, modulation of tumor metabolism, and induction of therapy resistance.

In promoting tumor proliferation and metastasis, B7-H3 primarily exerts anti-apoptotic effects via pathways such as Jak2/Stat3, PI3K/AKT, and E7/Rb, thereby enhancing tumor survival. Specifically, B7-H3 increases the phosphorylation of Jak2, then increasing the level of Stat3 phosphorylation [29]. By activating the pro-survival PI3K/AKT signaling pathway, B7-H3 up-regulates the level of BCL-2 proteins to enhance tumor cell proliferation and chemoresistance [30]. The effects of B7-H3 also lie in silencing the Rb mRNA and promoting the expression of E7 mRNA [31]. B7-H3 also activates pro-proliferative signaling through ERK and STAT3 and facilitates cell cycle progression by enabling tumor cells to bypass the G_1_/S checkpoint. Additionally, B7-H3 induces epithelial–mesenchymal transition (EMT) in tumor cells through the JAK2/STAT3 pathway, thereby increasing their invasive and metastatic potential. B7-H3 expression has been demonstrated to be positively correlated with IL-8, a cytokine known to promote metastasis [2,18]. Furthermore, B7-H3 enhances cancer cell invasion of surrounding tissues by up-regulating the activity of matrix metalloproteinases (MMPs) [32].

In the context of angiogenesis and tumor metabolism, B7-H3 promotes VEGF expression through the NF-κB and Tie-2 pathways, thereby facilitating tumor vascularization. Additionally, B7-H3 enhances reactive oxygen species (ROS) production through a distinct mechanism that stabilizes HIF-1α, thereby augmenting glycolysis. A study on breast cancer demonstrated that reduced B7-H3 expression leads to a decrease in glycolytic capacity, rendering tumor cells more susceptible to AKT/mTOR inhibitors. These findings establish a link between metabolism and B7-H3 [2,18]. In addition to glucose metabolism, B7-H3 is also associated with alterations in fatty acid metabolism. For instance, in glioma cells, genetic risk factors associated with fatty acid catabolism exhibit a significant correlation with the expression of B7-H3 [32].

B7-H3 contributes to the development of drug resistance through various mechanisms, including activation of the JAK2/STAT3 and PI3K/AKT pathways, upregulation of BRCC3 expression, and potentiation of the DUSP10 and MAPK cascades [2]. Furthermore, B7-H3 upregulates transmembrane 4 superfamily member 1 (TM4SF1) via an AKT-dependent mechanism, thereby antagonizing doxorubicin-induced G_2_/M arrest. It also enhances MEK signaling by activating major vault protein (MVP), promoting the conversion of more tumor cells into cancer stem cells (CSCs) [18].

### 2.3. Expression of B7-H3 in Tumor Tissues

#### 2.3.1. The Association Between B7-H3 and TME

The TME is a highly heterogeneous and complex system composed of tumor cells, various immune cells, cytokines, bone cells, and mineralized structures such as the extracellular matrix (ECM). It plays a crucial role in tumor initiation, progression, metastasis, and immune evasion [10]. Within the TME, several pro-tumor cell types have been identified, ref. [3] including M2-type tumor-associated macrophages (M2-TAMs), myeloid-derived suppressor cells (MDSCs), regulatory T cells (Treg cells), tumor-associated neutrophils (TANs), tumor-associated fibroblasts (TAFs), etc. Conversely, antitumor cells within the TME include [3] T cells, DCs, NK cells, B cells, M1-TAMs, C1q+ TAMs, etc. Additionally, excessive activation of the RANK-RANKL signaling pathway promotes osteoclast activation, exacerbating bone destruction in the surrounding tissue [33]. The intricate interactions among diverse cell types within the TME are an area of significant research interest. These cells form an immunosuppressive network through cytokine secretion or direct cell–cell interactions, driving tumor progression while also providing potential targets for immune intervention. A significant proportion of innate immune cells within the TME, including DCs, macrophages, NK cells, NKT cells, and γδT cells, also express immune checkpoint molecules. The antitumor activity of these cells can be reversed if these checkpoints are blocked [34]. Extensive studies have previously elucidated the role of the TME in OS, with these findings underscoring its pivotal role in disease progression and prognosis [35].

Given that the receptor for B7-H3 remains unclear, the role of B7-H3 in the TME remains largely unknown. It is evident that the elevated expression of B7-H3 in the TME is closely associated with the progression of OS. The immunoregulatory and non-immunoregulatory functions of B7-H3 are also closely tied to the TME. The known functions of B7-H3 include the inhibition of the proliferation of antitumor T cells, such as CD8^+^ T cells, CD4^+^ T cells, Th1 cells, and Th2 cells; the mediation of the polarization of TAMs towards the M2 phenotype, which is favorable for tumor growth; and the inhibition of the activation of NK cells, amongst others [21]. To better understand how B7-H3 functions within TME, several mechanisms are detailed here. As previously mentioned, B7-H3 can upregulate MMP expression in tumor cells, particularly MMP2 and MMP9. Invadopodia of tumor cells are rich in MMP2 and MMP9, and these protrusions serve as crucial structures enabling tumor cell invasion [36]. The PI3K/AKT/STAT3 pathway represents one mechanism through which B7-H3 upregulates MMPs. B7-H3 activates PI3K, which in turn activates AKT and promotes the expression of the transcription factor STAT3. STAT3 translocates to the nucleus, where it upregulates the expression of pro-invasive factors such as MMPs [36]. Furthermore, activated AKT can elevate HIF-1α protein levels via mTOR, enhancing anaerobic glycolysis in tumor cells and thereby promoting tumor growth [37]. The B7-H3 proteins, which are usually located on the tumor cell surface, bind to receptors on T cells, transmitting inhibitory signals [38]. B7-H3 receptors on other immune cells can also be bound by tumor cells, while elevated levels of IL-10, TGF-β1, and other cytokines in the TME create an immunosuppressive microenvironment [39]. In summary, B7-H3 interacts with the TME through multiple pathways, suppressing the activation and proliferation of antitumor cells such as CD4^+^ T cells while accelerating tumor cell growth [40]. Research on B7-H3 mechanisms has primarily been conducted in other tumor models, such as bladder cancer, gastric cancer, and cervical cancer. Based on the universality of natural laws, it is reasonable to assume that similar processes occur in OS. It remains crucial to emphasize that the interaction between B7-H3 and the TME constitutes a network of multiple interlinked pathways. These pathways influence each other rather than functioning in isolation.

#### 2.3.2. Expression of B7-H3 in OS and Its Association with Other Molecules

The high expression of B7-H3 in OS has been validated by many studies. In a Chinese cohort of 61 OS patients from 2004 to 2009, 91.8% of samples showed high B7-H3 expression [41]. A single-institution retrospective analysis from America assessed 156 sarcoma specimens. The study showed that 91% of samples had B7-H3 expression, and at least 80% of tumor cells per sample were positive. There were no significant differences among the results of various subtypes, including OS [42]. B7-H3 is usually expressed intracellularly, and can also exist in a soluble form extracellularly (soluble B7-H3 or sB7-H3). Intracellularly, B7-H3 is localized to the nucleus, cytoplasm, and plasma membrane. In recent years, there has been more and more evidence linking subcellular localization to tumor prognosis [43]. Increasing data indicate that sB7-H3 may be a potential biomarker of OS, and its level correlates with an unfavorable prognosis positively [44]. However, such studies specifically examining the subcellular localization of B7-H3 in OS cells remain limited, highlighting the need for further investigation.

The relationship among B7-H3, other immune checkpoints (e.g., PD-L1 and HVEM), and Circular RNAs (circRNAs) is yet to be fully elucidated. A study conducted on human and canine OS cell lines [45] revealed variable expression levels of B7-H3, PD-L1, and HVEM. Notably, PD-L1 expression was strongly correlated with both B7-H3 and HVEM expression, while the correlation between HVEM and B7-H3 was weak. Another study [46] analyzing samples from 35 patients with advanced OS demonstrated that some patients exhibited co-expression of multiple immune checkpoint molecules, with B7-H3 and CD47 being the most commonly co-expressed, while another clinical analysis of various sarcoma samples showed no significant correlation between B7-H3 expression and PD-L1 or PD-1 expression [42,47]. This discrepancy is further highlighted by a retrospective study of lung squamous cell carcinoma, which showed that B7-H3 expression was positively correlated with PD-L1 expression not only in tumor cells but also in various cells within the TME [48]. These conflicting findings underscore the need for further investigation into the association between B7-H3 and other immune checkpoint expression to gain a more comprehensive understanding of the complex OS TME.

CircRNAs have emerged as effective sponges for miRNAs, regulating immune checkpoint expression at the post-transcriptional level by competing with endogenous RNAs. For example, the circRNA variant hsa_circ0021347 has been shown to negatively correlate with B7-H3 expression [15]. Studies have indicated that the suppression of the B7-H3 in OS cells impedes their invasive and migratory capabilities, leading to alterations in the expression of 652 circular RNAs. Among the genes under consideration, hsa_circ0021347 was found to be significantly downregulated in OS tissues and cell lines. Furthermore, circRNAs can bind to RNA-binding proteins and serve as translation templates, modulating immune cells such as T cells, NK cells, and macrophages. These properties make circRNAs promising tools for developing immunotherapies, including vaccines and chimeric antigen receptor T cells (CAR-T) therapies [49]. However, clinical trials involving circRNAs are still limited [50]. The combination of circRNAs and B7-H3 represents a prospective direction for future research.

Furthermore, B7-H3 expression levels vary across different stages of OS progression. A study [51] using OS xenograft models in nude mice observed dynamic changes in B7-H3 expression throughout tumor progression. B7-H3 was relatively low during the early and mid-stages of tumor development but was significantly overexpressed in the late stage. Experimental findings also showed a substantial decline in CD3^+^ T cell numbers in the advanced stages of the disease. Additional studies utilizing mouse models have confirmed that B7-H3 expression escalated with OS progression [52]. This hypothesis was further supported by clinical sample studies, which revealed that elevated B7-H3 expression was associated with lung metastasis in OS [41,47].

## 3. Targeting B7-H3 Immunotherapies

A clinical observational study with an average follow-up duration of 5.1 years found that patients who developed infections after resection of primary OS exhibited higher 5-year survival rates and event-free survival rates [53]. This suggests that immune system stimulation may improve the outcomes of OS patients. In recent years, immunotherapy research has advanced significantly, and several therapeutic targets have been explored in OS treatment, including human epidermal growth factor receptor 2 (HER2), folate receptor (FR), interleukin-11 receptor α-chain (IL-11Rα), and GD2, among others. The effects of these targets vary, and there are many interesting experimental results [54,55,56,57,58]. B7-H3, which is highly expressed in OS cells, exerts an immunosuppressive effect by inhibiting T-cell proliferation and activation. Additionally, B7-H3 has been observed to promote osteosarcoma progression through its interaction with the TME and by influencing the expression of other molecules. Patients with high B7-H3 expression tend to have significantly shorter survival times and lower survival rates compared to those with low B7-H3 expression. These findings underscore the potential of targeting B7-H3 in immunotherapy to improve the prognosis and increase the 5-year survival rate of OS patients.

**Figure 3:**
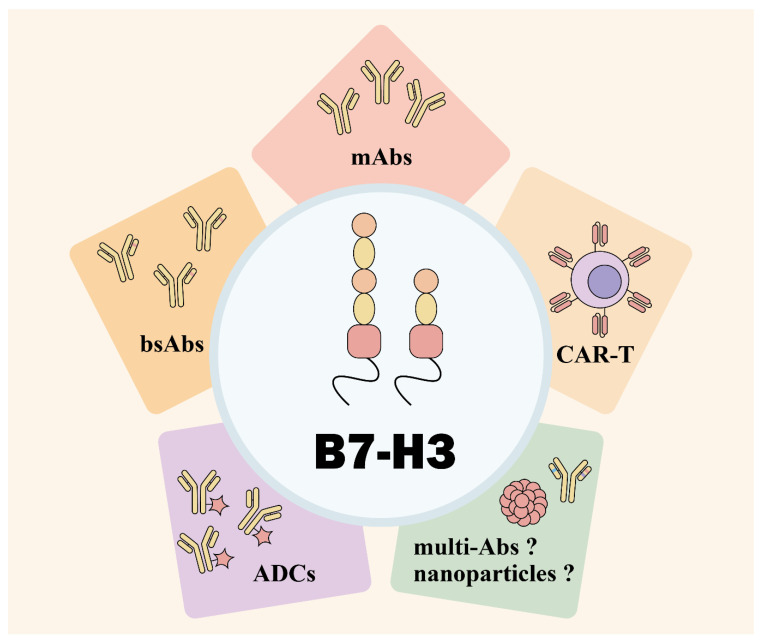
Targeting B7-H3 immunotherapies. The main strategies include mAbs, CAR-T therapy, and ADCs. Other potential approaches, such as bsAbs and multispecific antibodies, remain inadequately explored, which could also be combined with CAR-T and ADCs. Nanoparticles that take advantage of B7-H3 remain unexplored, either.

Immunotherapy strategies targeting B7-H3 are being actively explored across various solid tumors, with numerous agents currently undergoing preclinical or clinical trials [59]. The therapeutic potential of targeting B7-H3 immunotherapy has been extensively investigated, with several modalities being explored, including monoclonal antibodies (mAbs), adoptive cell therapy (ACT), antibody-drug conjugates (ADCs), and bispecific antibodies (bsAbs), amongst others (Figure 3). MAbs form the foundation of these immunotherapeutic approaches. CAR-T cells and ADCs both target tumor cells via B7-H3 antibodies and then exert their tumor-killing functions. In addition to targeting B7-H3, bsAbs incorporate new antigenic epitopes, thereby enabling them to simultaneously signal T cells, NK cells, macrophages, and other immune cells to attack tumors. The fundamental principles of these immunotherapy methods are well-established, and numerous studies have refined their application in clinical settings. The preclinical B7-H3-targeting immunotherapy strategies for OS are summarized in Table 1. On the other hand, we can also find that despite extensive interaction mechanisms between B7-H3 and TME, most immunotherapy strategies for OS have not fully leveraged this aspect. While many ICIs targeting conventional checkpoints like PD-1/PD-L1 and CTLA-4 have been developed, they have not demonstrated significant efficacy in OS [60,61]. Perhaps for this reason, emerging strategies primarily take advantage of the high expression of B7-H3 on OS cell surfaces. By utilizing anti-B7-H3 antibodies to direct drug delivery to tumor sites, these approaches exert their antitumor effects. Furthermore, advances in nanomaterials have contributed significantly to immunotherapy innovation. The utilization of nanomaterials has become an integral component of numerous tumor treatment strategies. However, research that combines nanoparticles with B7-H3 is limited, which may be one of the future directions for OS immunotherapy.

### 3.1. mAbs

Since their emergence in the 1970s, mAbs have been widely used in various medical fields [73]. These antibodies, which can bind to specific antigen epitopes, are monovalent, laboratory-prepared proteins derived from a single B cell clone. The selection of antigen epitopes determines the function of the mAbs. These specialized antibodies can exert their effects through several mechanisms, including enhancing immune system function, being conjugated with toxic drugs, and being labeled with radioactive isotopes [74]. More specifically, mAbs can enhance ADCC of NK cells or neutrophils, complement-mediated cytotoxicity, and phagocytosis of macrophages. Additionally, mAbs can be used to deliver drugs to target tumor sites, inhibit intracellular signaling pathways in tumor cells, and block tumor invasion and angiogenesis [17]. Reducing immunogenicity and improving antibody affinity are critical for enhancing mAb efficacy. As such, significant attention has been directed towards the B7-H3 molecule, which is highly expressed in OS. In a sense, the immunotherapy strategies for OS are derived from mAbs.

Currently, there are few mAbs targeting B7-H3 available for OS immunotherapy. Two mAbs targeting B7-H3, 8H9 (omburtamab) and MGA271 (enoblituzumab), have shown some efficacy in clinical trials against solid tumors. However, for various reasons, they have not been widely adopted for the treatment of OS [68,75]. In contrast, significant development has occurred in other immunotherapy strategies based on these antibodies, including CAR-T therapy.

Hagelstein et al. tried to reinforce the efficacy of B7-H3 mAbs by modifying the antibody Fc part [62]. This Fc optimization mAb (8H8_SDIE) has a higher affinity for the Fc receptor CD16 on immune effector cells like NK cells. In other words, 8H8_SDIE can achieve higher antitumor activity by enhancing NK cell-mediated ADCC. Compared with mAbs containing wild-type Fc, 8H8_SDIE can bind more effectively to NK cells and significantly enhance NK cell activation, degranulation, IFN-γ secretion, and NK effector molecule release, ultimately leading to more effective OS cell killing.

### 3.2. CAR-T

Chimeric antigen receptor-modified cell immunotherapy represents a prominent form of adoptive cell therapy. The principle behind ACT involves the extraction of immune cells from the patient or a donor, followed by in vitro modification, expansion, or activation, and reinfusion into the patient to enhance the immune system’s ability to combat disease. This strategy relies on engineering immune cells to specifically target and recognize diseased cells. CAR-T cell therapy is a novel strategy involving the genetic modification of a patient’s T cells to confer the ability to recognize specific tumor antigens. In 2017, the FDA approved CAR-T cell therapy for the treatment of pediatric acute lymphoblastic leukemia, where it has shown promising efficacy [76,77,78]. However, the application of CAR-T cells in solid tumors seems to be less effective, despite extensive research efforts [79,80]. The process of CAR-T cell therapy begins with the collection of a patient’s blood sample, followed by the isolation of T lymphocytes. After in vitro activation and expansion, the T cells are genetically modified using viral vector transfection to express chimeric antigen receptors (CARs) that recognize tumor-specific antigens. These engineered CAR-T cells are then reinfused into the patient to seek out and destroy tumor cells [17].

The efficacy of CAR-T therapy depends largely on the specificity of the epitope targeting of OS cells. This requires that the epitope be expressed at low levels in normal tissues while being highly expressed in metastatic tumors [81]. The design of CAR-T cells is influenced by the interaction between each component of the CAR, including the antigen recognition domains, structural elements, and costimulatory molecules [23]. The single-chain variable fragment (scFv) of mAbs forms the basis for the design of CAR-T cell antigen recognition domains. For example, mAb 376.96 and Enoblituzumab (MGA271) are specific antibodies against B7-H3. And CAR-T cells designed based on mAb 376.96 have shown promising efficacy in the immunotherapy of ovarian cancer [82,83]. MGA271, a well-known B7-H3 inhibitor, has demonstrated efficacy in treating renal cell carcinoma, bladder cancer, and glioma when fused with scFv to create CAR-T cells [84,85,86]. Majzner et al. constructed B7-H3 CAR-T cells using a humanized MGA271 single-chain variable fragment, which effectively inhibited OS growth [63]. The study further revealed that the efficacy of CAR-T cells is dependent on the density of B7-H3 expression within the tumor. These B7-H3 CAR-T cells exhibit limited activity against K562 xenografts with low B7-H3 expression (4750 molecules per cell), while inducing significant regression of MG63.3 osteosarcoma, which expresses higher levels of B7-H3 (93,544 molecules per cell). The results that CAR-T cells showed reduced activity against cells exhibiting low B7-H3 expression (e.g., most normal tissues) may potentially establish a safety window for treatment.

CAR-T cell development has gone through several generations. The first generation of CAR-T cells contained only antigen recognition domains. However, these cells often aged prematurely due to the absence of costimulatory signals, prompting the development of the second-generation (2G) design. The 2G-CAR-T introduced CD28 or 41BB as costimulatory domains and was successful, becoming the mainstream approach in current clinical applications. All FDA-approved CAR-T cell products are based on the second-generation design. While scientists explored combining the two costimulatory molecules, CD28 and 41BB, it was found that such a third-generation design was less effective than the second generation. Nguyen et al. discovered that expressing the 41BB ligand (41BBL) on CD8α/CD28-CAR-T cells could counteract the negative effects of incorporating 41BB into the CAR structure [64], thereby enhancing the ability of CAR-T cells to kill metastatic OS cells.

**Figure 4:**
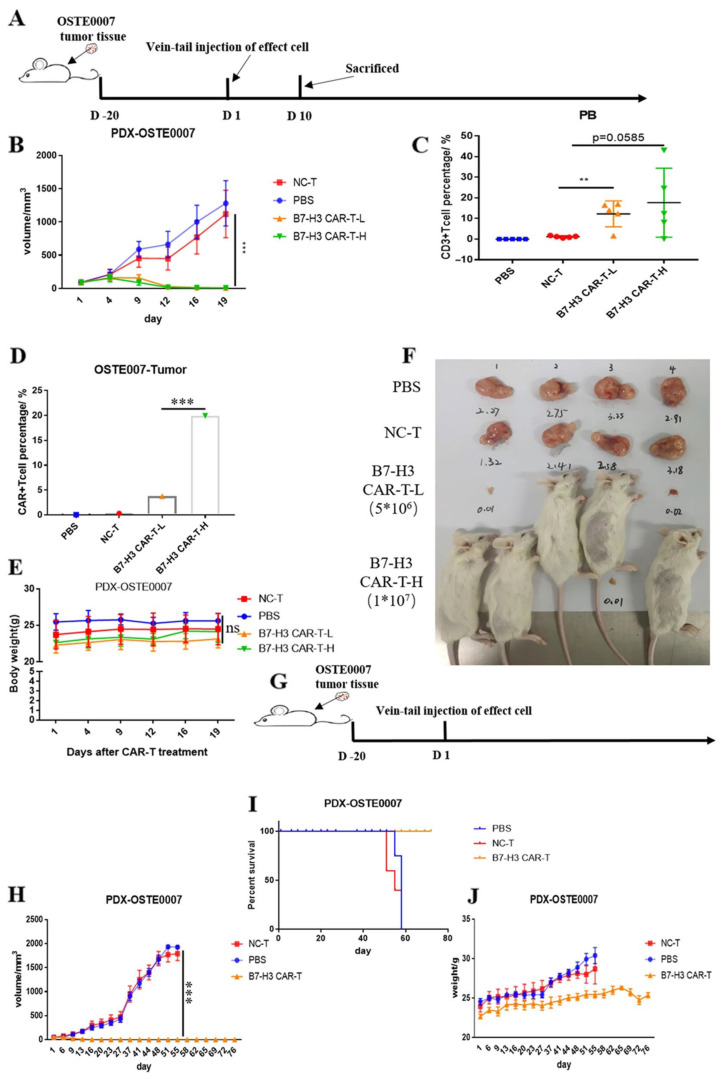
Anti-tumor efficacy of B7-H3 CAR-T cells against PDX models of osteosarcoma in vivo. (**A**,**G**) Schematic representation of experimental design. (**B**,**H**) Tumor growth curves of mice treated with B7-H3 CAR-T cells or control T cells. (**C**) Detection of CD3^+^ T cells in peripheral blood at day 10 post-infusion. (**D**) Lack of CAR-T cell infiltration within tumors at day 10 post-infusion. (**F**) Representative images of tumor masses from mice treated with B7-H3 CAR-T cells, NC T cells, or PBS. (**E**,**J**) Body weight changes in mice during treatment. (**I**) Kaplan–Meier survival curves of PDX-OSTE0007 models. (mean  ±  SEM; ns not significant *p*  >  0.05, ** *p*  <  0.01, *** *p*  <  0.001). Reproduced with permission from Ref. [65]. Copyright © 2022, The Author(s).

Recent research has also demonstrated progress in third-generation CAR-T cells, which target B7-H3. Zhang et al. constructed B7-H3 CAR-T cells that showed effective anti-OS activity (Figure 4) [65]. These cells contain a CD8 leading sequence, a CD8 hinge and transmembrane sequence, and the intracellular signaling domains of 4-1BB, CD28, and CD3 in tandem. In vitro cytotoxicity assays and in vivo xenograft models (PDX) revealed that these CAR-T cells could efficiently recognize and eliminate B7-H3-positive OS cells, releasing cytokines such as IFN-γ and TNF-α in the process. In PDX models, CAR-T cells significantly inhibited tumor growth and prolonged mouse survival time. These results suggest that B7-H3-targeted 3G-CAR-T cells hold considerable potential for OS treatment.

A major limitation of CAR-T cell therapy is the insufficient migration and accumulation of CAR-T cells at the tumor site. Given the crucial role of chemokines in promoting immune cell homing and migration, enhancing chemokine-mediated migration is a promising way to improve CAR-T cell targeting. Based on this rationale, two promising receptors—CXCR2 and CXCR6—were discovered to enhance B7-H3 CAR-T cell homing ability, functionality, and metabolic fitness against OS [66,67]. In one study [66], researchers evaluated the efficacy of B7-H3.CAR-T cells expressing CXCR2 and CXCR6, and the results show that these T cells exhibited ligand-specific enhanced migration over the control group. In a metastatic OS model, animals treated with CXCR2- or CXCR6-B7-H3 CAR T cells exhibited significantly prolonged survival. In another study [67], researchers constructed B7-H3 CXCR2 (BC2) CAR-T cells, which showed stronger homing ability, a unique gene expression profile, enhanced functionality, and superior metabolic fitness. T cells’ activation, differentiation, and functionalization require high energy, and metabolic antagonism in the TME weakens these processes [87,88]. Therefore, enhancing the metabolic adaptability of CAR-T cells is a promising approach. These findings suggest that targeting chemokines could improve the effectiveness of CAR-T cell therapy for OS and provide new insights for clinical applications.

**Figure 5:**
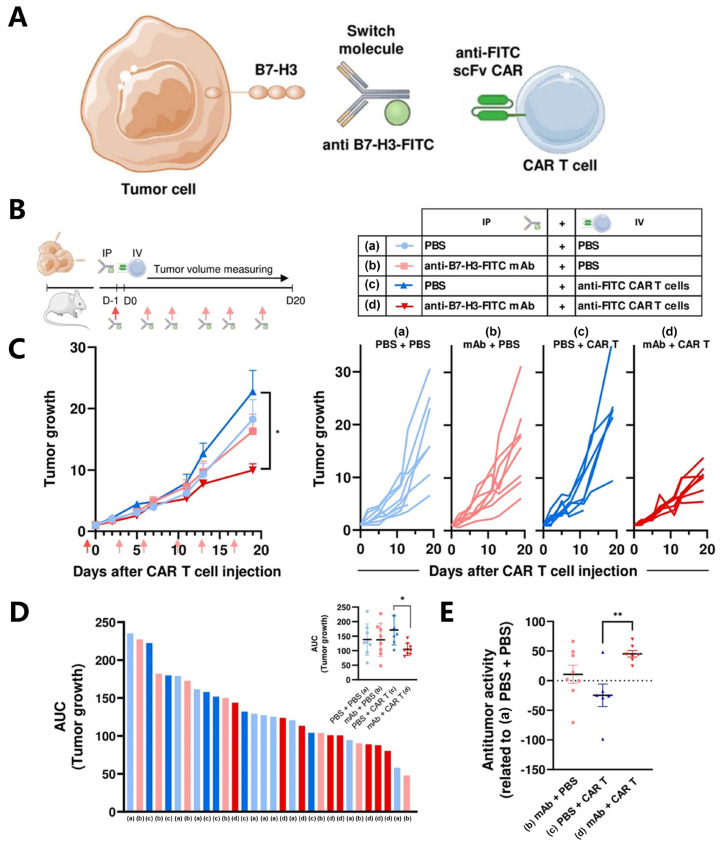
In vivo efficacy of CAR T cell treatment in OS tumors. (**A**) Schematic of the switchable CAR-T strategy using an anti-B7-H3 mAb conjugated with FITC and anti-FITC CAR-T cells. (**B**) Experimental design: NSG mice bearing 143B tumors received intraperitoneal injection (IP) of PBS (blue) or anti-B7-H3-FITC mAb (100 μg; red) on day −1. On day 0, mice were administered intravenously (IV) with PBS (light blue, (**a**); light red, (**b**)) or 5 × 10^6^ CAR-T cells (dark blue, (**c**); dark red, (**d**)). PBS or anti-B7-H3-FITC mAb (50 μg; red arrows) was subsequently administered every 3–4 days. The arrows indicate the time of injection. (**C**) Tumor growth curves under different conditions, shown as mean ± SEM (left) and individual values (right). * *p* < 0.05, one-way ANOVA with Tukey post hoc test. (**D**) Areas under the curve (AUC) of tumor growth in individual mice (left) and group means ± SD (inset). * *p* < 0.05, one-way ANOVA with Tukey post hoc test. (**E**) Relative antitumor activity (mean ± SD) of each treatment group compared with the PBS + PBS (A) control. ** *p*  <  0.01 by one-way ANOVA with Tukey post hoc test. Reproduced with permission for Ref. [69]. Copyright © 2023, The Author(s).

An alternative approach to enhance CAR-T therapy is adding a “switch” to CAR-T cells. Anti-B7-H3 mAbs were conjugated with FITC molecules to construct CAR-T cells that target FITC molecules (Figure 5) [69]. FITC serves as the switch molecule that activates the CAR-T cells, enabling them to target B7-H3-positive OS cells. In vitro experiments have shown that, in the presence of anti-B7-H3-FITC mAb, anti-FITC CAR-T cells can produce cytokines and effectively kill B7-H3-positive OS cells. In vivo experiments showed that anti-B7-H3 mAb can penetrate tumors and bind to B7-H3-positive tumor cells. This enables anti-FITC CAR-T cells to migrate to tumor sites and exert antitumor effects. This CAR-T cell approach has the advantage of being able to flexibly control CAR-T cell activation and function, thereby maximizing the avoidance of off-target toxicity. However, it is also important to note that the immunogenicity of FITC raises disputes over the clinical translation. The concept of “switch” is quite promising, and using non-immunogenic peptides to construct such switchable CAR-T cells is of great significance for further study.

Another strategy to mitigate CAR-T–associated toxicities is real-time monitoring of CAR-T cell distribution in vivo. Murty et al. developed an imaging and suicide ablation system that enables visualization of CAR-T cell trafficking and proliferation during clinical application while simultaneously providing a safety mechanism to minimize potential toxicities [89]. In this system, a mutated HSV1-tk (sr39tk) gene was incorporated into B7-H3 CAR-T cells. The antitumor efficacy of B7-H3 CAR-T cells remained largely unchanged after sr39tk modification, while the engineered cells became traceable by PET/CT imaging in an orthotopic OS model. After eight days, when 5 × 10^6^ B7-H3-sr39tk CAR T cells and 5 × 10^6^ sr39tk T cells were injected, [^18^F]FHBG PET/CT images demonstrated visibly higher uptake in the tumor of B7-H3-sr39tk CAR T cells–treated mice, while the uptake of muscle showed no obvious differences. Additionally, the sr39tk could serve as a suicide switch through treatment with ganciclovir. PET imaging of B7-H3-sr39tk CAR T cells confirmed complete tumor ablation with intraperitoneal ganciclovir administration. Such image-guided CAR-T platforms offer a promising avenue for clinical translation, as they not only enhance safety surveillance but may also support individualized dosing, early toxicity prediction, and adaptive treatment adjustments in future OS immunotherapy trials.

As CAR-T cell strategies are constantly improving, more effective models are also needed to evaluate and predict their clinical safety and efficacy. Currently, most efficacy prediction models are based on orthotopic OS in xenografts, which often fail to recapitulate the metastatic behavior that represents the greatest challenge in clinical management. To address this limitation, Talbot et al. developed a novel spontaneously metastasizing orthotopic OS model and utilized it to predict the safety and efficacy of B7-H3 CAR-T [90]. This model takes advantage of LM7 OS cells expressing firefly luciferase (LM7.ffLuc), which allows non-invasive monitoring of B7-H3 CAR-T cell trafficking to tumors. Compared to the method of directly injecting tumor cell suspensions into the tibia of mice, the method of implanting LM7.ffLuc in a collagen mesh into the tibial defect of mice mimics the spontaneous lung metastasis of OS more ideally.

Addressing these challenges, including the mitigation of the immunosuppressive effects of the TME, the enhancement of the targeting specificity of CAR-T cells while reducing off-target toxicity, the improvement of penetration into tumor tissue, the evasion of tumor immune escape, and the mitigation of downregulation of target antigens, holds significant value for advancing the clinical translation of CAR-T cell therapy. At present, the majority of CAR-T research is focused on solid tumors or sarcomas. CAR-T therapy targeting OS is also a promising area of study. However, this assertion is contingent upon further in-depth analysis of the OS-specific TME. Furthermore, cell therapy strategies that incorporate CAR modifications, such as CAR-NK and CAR-M therapies, have not demonstrated substantial progress in OS research. While these therapies theoretically have the potential to address the limitations of CAR-T therapy, they remain far from clinical application [3].

### 3.3. ADCs

ADCs represent a rapidly evolving cancer treatment strategy that integrates the targeting precision of mAbs with the therapeutic potency of chemotherapy drugs. This integration facilitates the precise delivery of toxic drugs to tumor tissues, thereby expanding the treatment window and enhancing therapeutic efficacy. Each ADC consists of three main components—an antibody, a linker, and a payload. Growing evidence suggests that the efficacy of a given ADC depends on how its antibody, linker, and payload components interact with the tumor and the microenvironment [91]. Chemotherapy is the conventional approach to OS and is an indispensable part of the management for the malignant tumor. Standard agents include doxorubicin, cisplatin, methotrexate, ifosfamide, etc. [92]. Over the years, chemotherapy drugs have been continuously refined, but adverse effects have persisted. Scientists are striving to strike a balance between maximizing therapeutic efficacy and minimizing harm to patients’ well-being. The advent of ADCs has tipped the balance in a positive direction, making immunotherapy strategies a powerful ally of chemotherapy drugs [93].

Kendsersky et al. investigated the safety and efficacy of m276-SL-pyrrolobenzodiazepine (PBD) in pediatric solid malignancy xenograft models. M276-SL-PBD is a PBD—armed ADC against B7-H3 [70]. M276 has been shown to specifically recognize and bind to B7-H3 when expressed on the surface of tumor cells. The drug-antibody complex is internalized by tumor cells and enters the cell interior. Within the cell, the SL linker arm is cleaved, releasing PBD. PBD has been shown to bind to the DNA of tumor cells, resulting in the induction of DNA damage and subsequent tumor cell death. Except for one model, the results in OS models showed that the median time-to-event in the treatment group was prolonged by at least 40 days compared to the control group, and the tumor volume regression exceeded 90%, which demonstrated that m276-SL-PBD exhibited significant antitumor activity. MGC018 is a duocarmycin-based humanized ADC targeting B7-H3, displaying profound antitumor activity in many solid cancers like lung cancer and melanoma [94]. Kurmasheva et al. evaluated MGC018 in OS models and achieved satisfying results [71].

HS-20093, also known as CSK’227, is the latest breakthrough in ADCs [95,96]. As a novel B7-H3-targeted ADC, it is composed of a fully humanized anti-B7-H3 monoclonal antibody that is covalently linked to a topoisomerase inhibitor payload. This enables the drug to specifically induce apoptosis in tumor cells that express B7-H3. In January 2025, the FDA granted HS-20093 breakthrough therapy designation for the treatment of relapsed or refractory (R/R) OS in adult patients. YL201 is a novel B7-H3 targeting ADC, leveraging a TME activable linker-payload platform, coupled with a novel topoisomerase 1 inhibitor via a protease-cleavable linker [97]. MHB088C, DS-7300a are also novel B7-H3 targeting ADCs loaded with DNA topoisomerase 1 inhibitor [98,99]. They all exhibit promising results in clinical trials, yet there are not many experiments that are conducted in OS models.

In conclusion, ADCs represent the most rapidly evolving pharmaceutical concept in the domain of cancer treatment. The broad spectrum of target options and drug payloads endows ADCs with considerable scalability, such that each target optimization and new drug development unlocks new potential for ADCs. Paul Ehrlich’s early 20th-century “magic bullet” idea of targeting drugs to tumor cells laid the foundation for the development of contemporary ADCs [100,101]. Early ADCs demonstrated substantial toxicity without manifest clinical efficacy [91]. Nowadays, the development of ADCs is a flourishing field of research. The advent of rapid advancements in the domain of synthetic biochemistry has had a considerable impact on the development of ADCs. Presently, there are more than ten ADCs that have received FDA approval for tumor treatment; however, only a small number of these drugs target B7-H3, and even fewer are utilized for the treatment of OS. In the context of immunotherapy for OS, there remains significant potential for further exploration with ADCs that has yet to be realized.

### 3.4. bsAbs

MAbs and their derivatives, CAR-T cells, and ADCs show exciting prospects. The idea of optimizing antibodies further has led to the development of bsAbs. Furthermore, trispecific and multispecific antibodies, as well as CAR-T and ADC therapies based on these, also have possibilities to be explored. This dual-targeting capability has enabled the application of bsAbs in treating a variety of malignancies. For instance, blinatumomab is applied to treat relapsed/refractory B-cell acute lymphoblastic leukemia [102], while glofitamab and epcoritamab are used in the treatment of large B-cell lymphoma [103,104].

**Figure 6:**
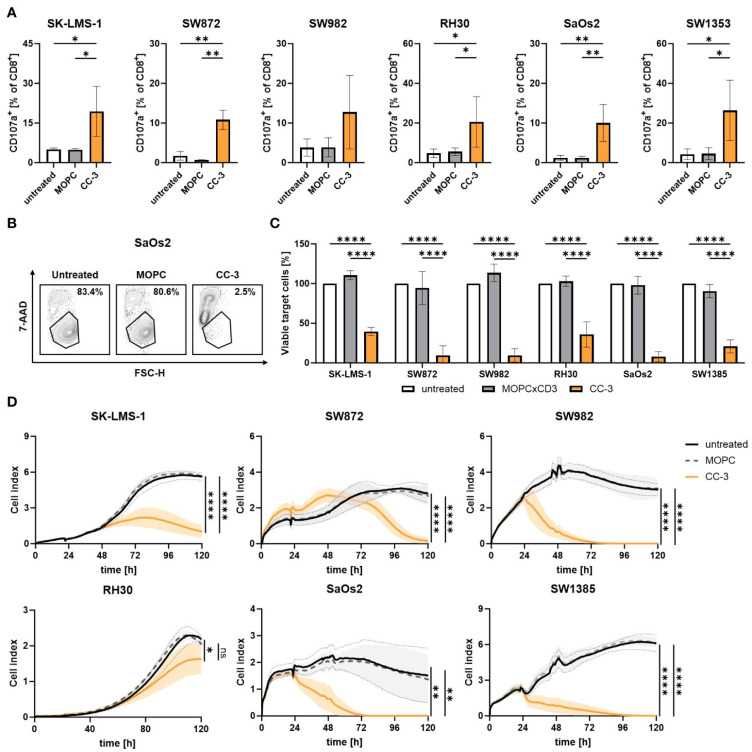
CC-3 enhances the effector function of cytotoxic T cells against sarcoma cells. Peripheral blood mononuclear cells (PBMCs, n = 4) were cocultured with sarcoma cell lines at an effector-to-target (E–T) ratio of 5:1 in the presence or absence of CC-3 (1 nM) or MOPC isotype control. (**A**) Degranulation of CD8^+^ T cells, measured by CD107a expression after 4 h of coculture. (**B**,**C**) Tumor cell killing after 72 h, quantified by flow cytometry; cell numbers from untreated controls were normalized to 100%. (**D**) Sustained cytotoxic activity of PBMCs (n = 4) against sarcoma cells monitored in real time using the xCELLigence system over 120 h. The values presented are means ± SD (* *p* < 0.05, ** *p* < 0.01, **** *p* < 0.0001). Reproduced with permission from Ref. [72]. Copyright © 2024 Holzmayer, Liebel, Hagelstein, Salih, and Märklin.

Holzmayer et al. reported a B7-H3xCD3 bispecific antibody in IgG-based format (termed CC-3) and evaluated it in multiple sarcoma subtypes that included OS (Figure 6) [72]. The synthesis of CC-3 is based on the previous studies, which used CC-3 for the treatment of gastrointestinal cancers [105,106]. Compared to MOPC (using MOPC as a control antibody to construct CAR-T cells), CC-3 elicited robust T cell responses against multiple sarcoma subtypes, driving significant activation accompanied by cytokine and effector molecule release. Furthermore, it enhanced T cell proliferation and differentiation, leading to the generation of memory T cells. Notably, CC-3 also induced potent target cell lysis in a target cell-restricted manner.

### 3.5. Comparative Evaluation of Therapeutic Strategies

The four major strategies—mAbs, CAR-T, ADCs, bsAbs—possess their own advantages and deficiencies. Mabs can intervene in the signaling pathways mediated by B7-H3, thereby modulating the suppressive TME. However, few anti-B7-H3 antibodies are applied in clinical practices, perhaps for the underlying reasons that mAbs which target other immune checkpoints achieve low efficacy in solid tumors. CAR-T, ADCs, and bsAbs are based on mAbs, extending their functions. CAR-T research has been the most extensively studied, largely due to its modifiability. Scientists can employ various approaches to endow CAR-T with richer functionalities, representing one of its most prominent advantages. While CAR-T has achieved significant success in treating lymphoid and hematopoietic system tumors, its efficacy in solid tumors like OS remains unconfirmed. The primary advantage of ADCs lies in their ability to carry drugs, with B7-H3 targeting maximizing therapeutic efficacy. However, B7-H3 expression is not completely exclusive to tumor tissues; the off-target toxicity remains a significant challenge. Though research on bsAbs is relatively limited, this represents a promising area of development. Both CAR-T and ADCs can be further optimized through bsAbs. Overall, complementary and combined strategies represent a crucial future development direction.

Table 2 summarizes the clinical trials of immunotherapies targeting B7-H3 for OS. Comparing the clinical trials and preclinical studies, we can find that immunotherapy approaches targeting B7-H3 are advancing rapidly, whereas clinical trials for OS remain scarce. Most preclinical studies have yet to translate into clinical trials. This indicates significant potential for the clinical translation of relevant immunotherapy strategies, while it also suggests a long road ahead before these approaches can be applied to the clinical treatment of OS.

## 4. The Application of Nanoparticles

Recent years have seen continuous development in nanotechnology, with the result that nanomaterials have demonstrated broad application prospects in tumor immunotherapy. The delivery of immunotherapy drugs, including B7-H3 antibodies, siRNAs, and small-molecule inhibitors, can be facilitated by the utilization of nanomaterials. This approach enables the targeted delivery of these drugs to tumor sites, thereby enhancing treatment efficacy and reducing adverse effects. Moreover, nanomaterials show promise in facilitating a range of therapeutic modalities, including photodynamic therapy (PDT), chemodynamic therapy (CDT), photothermal therapy (PTT), and the synergy between chemotherapy and immunotherapy [107] is a noteworthy consideration. The integration of nanomaterials into OS immunotherapies facilitates the effective circumvention of the limitations associated with conventional strategies [108]. Specific applications encompass two aspects: first, developing advanced drug delivery systems; second, targeting immune cells or organs to reshape the TME [12]. The unique physicochemical properties of nanomaterials offer scientists extensive opportunities to develop multifunctional nanoplatforms, which is quite promising to touch a brighter future.

Organic nanomaterials, represented by metal–organic frameworks (MOFs), have demonstrated significant advantages in drug delivery, enabling precise control over drug release. Inspired by the design of CAR-T cells, a study developed a biomimetic targeted co-delivery system, utilizing an anti-B7-H3 macrophage membrane to enhance the targeting capability of these nanoparticles [109,110]. Two drugs—CDK4/6 inhibitors and poly ADP-ribose polymerase (PARP)—were loaded in MOFs to impair OS cells’ DNA repair capacity. And the anti-B7-H3 bio-membrane largely amplified the tumor-suppressive effect. In addition, experiments in tumor-bearing mice models suggest that the combination of this co-delivery system and PDT could increase CD8^+^ T cell infiltrations in TME. This study indicates that the nanoparticle system confers drugs with greater characteristics, while the specific targeting of B7-H3 maximizes the therapeutic efficacy in OS treatment.

**Figure 7:**
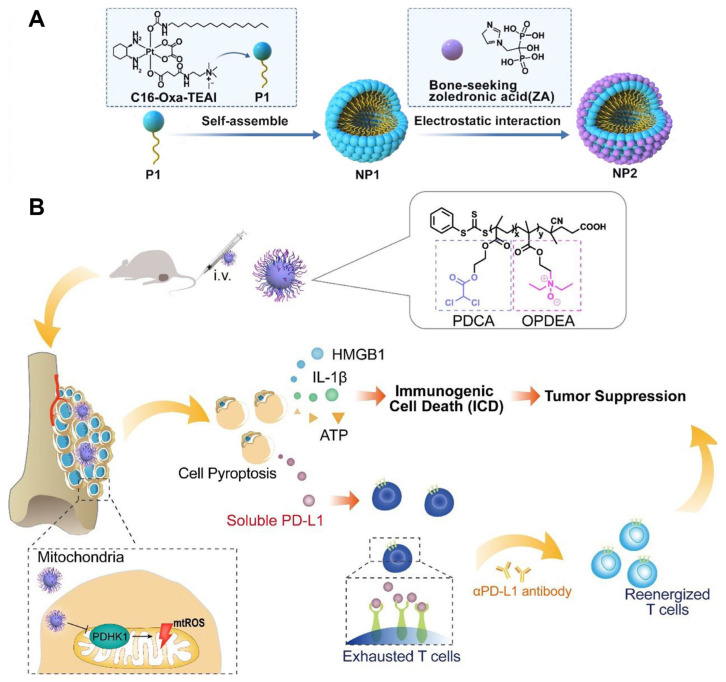
(**A**) The synthesis of NPs, which have the properties of pH-responsive and glutathione (GSH)-responsive and high affinity for tumors. Reproduced with permission from Ref. [111]. Copyright © 2023 Published by Elsevier Ltd. (**B**) Schematic illustration showing how mitochondria-targeted OPDEA-PDCA induces pyroptosis in OS cells and how combination treatment with OPDEA-PDCA and an anti-PD-L1 monoclonal antibody enhances antitumor activity. Reproduced with permission from Ref. [112]. Copyright © 2022 American Chemical Society.

Although nanotechnology has made rapid progress, B7-H3-targeted nanoparticles are quite rare. Figure 7 exhibits two approaches targeting other immune checkpoints for the treatment of OS. Li et al. reported dual-responsive nanoparticles (NP2), which can enhance the antitumor efficacy and stimulate the immune response simultaneously [111]. These nanoparticles exhibit both pH- and glutathione (GSH) -responsiveness, along with high affinity for tumors. Upon accumulation in tumor tissue, the acidic TME induces NP2 to release positively charged nanoparticles (NP1) and negatively charged zoledronic acid (ZA), which can then trigger a series of reactions. The NP2 casts light on the possibility of combining immunotherapy with chemotherapy. In a related study, Jin et al. [112] synthesized a mitochondria-targeting polymer micelle (OPDEA-PDCA). This micelle has been observed to impede the function of pyruvate dehydrogenase kinase 1, a process that has been demonstrated to induce oxidative stress within the mitochondrial compartment. This effect has the potential to induce immunogenic pyroptosis in OS cell lines. Furthermore, the study revealed that while OPDEA-PDCA induces pyroptosis, it concomitantly increases the secretion of PD-L1 molecules. This finding suggests that a combination of PD-L1 inhibitors with treatment could significantly enhance the efficacy of immunotherapy. This finding suggests a potential avenue for developing B7-H3-related micelles to enhance immunotherapy.

It is quite exciting that there are various ways to develop nanotechnology-based immunotherapy for OS. The utilization of nanoparticles confers a multitude of benefits, including enhanced targeting, reduced off-target toxicity, and augmented drug potency. Apart from what is mentioned above, specific approaches include leveraging liposomes, hydrogels, chitosan, nano-oxides, nanoalloys, carbon nanotubes, micelles, dendritic macromolecules, nanocapsules, and exosomes [12]. Besides the advantages of nanotechnology-based immunotherapy, limitations remain. These include challenges in controlling the biodistribution and clearance of nanoparticles. Additionally, issues like penetrating the blood-tumor barrier and decreasing the toxicity to vital organs are also crucial aspects. It is also necessary to emphasize that the advent of emerging nanotechnology has not yet fully capitalized on the potential of B7-H3 in the treatment of OS. If further study could combine nanoparticles with B7-H3 appropriately, the immunotherapies for OS must be promoted tremendously.

## 5. Conclusions and Future Directions

OS represents the most prevalent primary malignant tumor among adolescents, yet conventional treatments have had limited success in significantly improving patient outcomes. Immunotherapy, as one of the most rapidly developing emerging fields, holds considerable promise for enhancing long-term survival rates in OS patients. Extensive research has demonstrated that B7-H3 is overexpressed in OS, as well as other solid tumors, and its expression is positively correlated with tumor metastasis and poor prognosis. Elevated B7-H3 levels are often accompanied by a decrease in tumor-infiltrating T lymphocytes, which are the predominant antitumor effector cells. This is a critical mechanism by which tumors evade the immune system. This review has discussed the expression and regulation of B7-H3, as well as its dual role in both immunoregulatory and non-immunoregulatory functions. While B7-H3 mRNA is widely expressed in both immune and non-immune tissues, B7-H3 protein is predominantly localized to tumor cells. These characteristics suggest that B7-H3 may have significant potential as an immunotherapy target, which has sparked increased scientific interest.

In the field of tumor treatment, a variety of B7-H3-based immunotherapy strategies have been developed, showing promising evidence of their efficacy and potential. These strategies for OS primarily include mAbs, CAR-T therapy, ADCs, and bispecific antibodies, with the majority of research focused on CAR-T. It can be argued that mAbs serve as the foundation for most immunotherapy strategies, enabling targeted delivery of drugs or immune cells to tumor cells while minimizing damage to normal tissues. While the fundamental principles of these approaches are straightforward, optimizing and enhancing them is a complex challenge. The majority of research endeavors are centered on this area, including expressing chemokine receptors on the surface of CAR-T cells and incorporating switches within CAR-T cells. These ongoing innovations highlight that an optimal solution has yet to be found. The development of multispecific antibodies, which are based on the concept of bispecific antibodies, represents a promising avenue for further optimization of ADCs and CAR-T cells. CAR-M and CAR-NK have also demonstrated encouraging outcomes in the treatment of other tumors; however, their application in OS therapy is still largely unexplored. In parallel, nanotechnology has gained considerable attention in medical research and offers novel tools for advancing therapeutic strategies. This technological advancement has significantly contributed to the evolution of treatment methodologies and has furnished healthcare professionals with sophisticated instruments to combat diseases that were formerly incurable. Notably, studies have demonstrated that the combination of immune checkpoint molecules with nanoparticles can yield significant antitumor effects. However, the specific role of B7-H3 in this context remains poorly investigated. Collectively, these findings indicate that there is considerable potential for the development of diverse immunotherapy approaches for OS.

It is also important to note that the receptor for B7-H3 has not yet been identified, which limits our understanding of its biological functions and mechanisms within the TME. Though the majority of immunotherapeutic strategies are based on the phenomenon that B7-H3 is specifically expressed in OS, further exploration of the interactions between B7-H3 and various cell types in the TME also makes a difference. A better understanding of the biological function will help scientists to improve and advance related immunotherapy strategies with the aim of achieving higher antitumor efficacy. In addition, from the perspective of clinical translation, the high heterogeneity and immunosuppressive characteristics of TME pose challenges for translating immunotherapy into clinical practice, though it offers numerous potential avenues for immunotherapy at the same time. Future research on B7-H3-targeted immunotherapies for OS should focus on several key priorities. These include identifying additional receptors that could enhance targeting efficacy, improving precision in drug delivery to tumor tissues, and reducing off-target toxicity. Addressing the immune-suppressive properties of the TME and developing strategies to better activate the body’s immune system while preventing tumor immune escape are also critical. Moreover, the integration of combinatorial immunotherapy approaches, along with innovations in nano-delivery systems, may help overcome the clinical challenges posed by the OS TME and improve therapeutic outcomes. Looking forward, with continued progress in preclinical studies and clinical trials, B7-H3-targeted therapies could eventually be integrated into OS treatment pipelines, offering new therapeutic options for this challenging disease.

## Figures and Tables

**Table 1:** Preclinical B7-H3-targeting immunotherapy strategies for OS.

Strategy	Conception	Methods	Year	Reference
mAb	By modifying the Fc segment gene of B7-H3 mAbs, its affinity with NK cells can be enhanced, thereby enhancing NK-antibody-dependent cellular cytotoxicity (ADCC).	Generation of 8H8_SDIE was carried out by chimerization (human immunoglobulin G1/κ constant region) of the anti-B7-H3 mAb 8H8 and Fc-optimization for mAbs.	2022	[62]
CAR-T	Based on humanized MGA271 scFv.	The MGA271 scFv was integrated into a retroviral vector with the CD8α hinge/transmembrane domain, 4-1BB costimulatory domain, and CD3ζ signaling domain through codon optimization, and then transduced into T cells.	2019	[63]
CAR-T	41BB ligand (41BBL) is expressed on the surface of traditional 2G-CAR-T.	The effects of integrating 41BB into the CAR structure and expressing 41BBL on the surface of CAR-T cells were compared, with the latter enhancing the killing of OS cells.	2020	[64]
CAR-T	The third-generation CAR-T, which targets B7-H3.	a CD8 leading sequence, a CD8 hinge and transmembrane sequence, and the intracellular signaling domains of 4-1BB, CD28, and CD3ζ in tandem.	2022	[65]
CAR-T	Target chemokines to enhance T cell homing ability, and CXCR2 and CXCR6 are identified as two effective receptors.	Use lentiviral transduction to express the CXCR2 or CXCR6 gene, followed by retroviral transduction to express the B7-H3. CAR gene, thereby achieving dual modification.	2024	[66]
CAR-T	OS expresses IL-8 (CXCL8). Enhances the antitumor effect of B7-H3 CAR-T by expressing its receptor CXCR2 on T cells.	Extract RNA from neutrophils that naturally express CXCR2. Convert RNA to cDNA by reverse transcription, and the CXCR2 sequence is PCR amplified and inserted into the B7-H3 CAR retroviral construct.	2024	[67]
CAR-T	CXCR2 co-expressing.	Extract CXCR2 RNA from neutrophils and construct the B7-H3-CXCR2-CAR. Characterize its properties in canine OS cell lines.	2024	[68]
CAR-T	Using B7-H3-FITC mAb as a switch molecule, this molecule can target OS. FITC CAR-T then migrates to the tumor tissue by targeting the switch molecule.	Obtain anti-B7-H3 mAb through 8H9 hybridoma, and it is conjugated with FITC. FITC CAR-T contains a fully human anti-FITC scFv, an IgG4 hinge-CH2-CH3 spacer, a CD28-transmembrane domain, a 4-1BB/CD3z signaling domain, and a cell-surface human EGFRt tag.	2023	[69]
ADC	Use the m276 antibody platform and covalently link pyrrolobenzodiazapine (PBD) with a cleavable valine-alanine linker.	Use m276-SL, a modified version of the fully human m276 IgG1 antibody engineered to contain four mutations in the Fc domain, to construct the m276-SL-PBD ADC.	2021	[70]
ADC	MGC018, a humanized ADC targeting B7-H3 based on the DNA alkylating agent duocarmycin.	Test MGC018 in several models of pediatric solid tumors, including OS.	2021	[71]
bsAbs	Bispecific B7H3xCD3 antibody, termed CC-3. CC-3 simultaneously targets the B7-H3 molecule on tumor cells and the CD3 molecule on T cells.	CC-3 is being evaluated in a phase 1 clinical trial for the treatment of metastatic cancer. This study evaluated the efficacy of CC-3 for the treatment of multiple sarcoma subtypes.	2024	[72]

**Table 2:** Clinical trials of immunotherapies targeting B7-H3 for OS.

Drug Type	Trail Number	Study Title	Intervention/Treatment	Trial Phage	Status
mAbs	NCT02982941	Enoblituzumab (MGA271) in Children with B7-H3-expressing Solid Tumors	Enoblituzumab	Phase 1	Completed
CAR-T	NCT04897321	B7-H3-Specific Chimeric Antigen Receptor Autologous T-Cell Therapy for Pediatric Patients with Solid Tumors (3CAR)	Fludarabine + Cyclophosphamide + MESNA + B7-H3 CAR T cells	Phase 1	Recruiting
	NCT04483778	B7H3 CAR T Cell Immunotherapy for Recurrent/Refractory Solid Tumors in Children and Young Adults	Second generation 4–1BBζ B7H3-EGFRt-DHFR (selected) + A second generation 4–1BBζ CD19-Her2tG + Pembrolizumab	Phase 1	Active, not recruiting
	NCT06500819	Autologous B7-H3 Chimeric Antigen Receptor T Cells in Relapsed/Refractory Solid Tumors	B7-H3CART Dose (Intravenous)	Phase 1	Recruiting
	NCT07222735	Hypofractionated Radiation in Combination with B7-H3-CAR T Cells for Pediatric Patients With Relapsed/Refractory Sarcomas	Fludarabine + Cyclophosphamide + B7-H3-CAR T Cells + Radiation Therapy	Phase 1	Not yet recruiting
ADC	NCT05830123	ARTEMIS-002: HS-20093 in Patients with Relapsed or Refractory Osteosarcoma and Other Sarcomas	HS-20093	Phase 2	Recruiting
	NCT06699576	ARTEMIS-103: Phase 1b Study of HS-20093 Combinations in Patients with Bone and Soft Tissue Sarcoma.	HS-20093 + Anlotinib + Epirubicin + Adebrelimab	Phase 1	Not yet recruiting

## Data Availability

No new data were created or analyzed in this study.

## Abbreviations

The following abbreviations are used in this manuscript:

OS	Osteosarcoma
TME	Tumor microenvironment
mAbs	Monoclonal antibodies
CAR-T	Chimeric antigen receptor T cells
ADCs	Antibody-drug conjugates
bsAbs	Bispecific antibodies
ICIs	Immune checkpoint inhibitors
IgC domains	Immunoglobulin constant-like domains
IgV domains	Immunoglobulin variable-like domains
APCs	Activated antigen-presenting cells
miRNAs	MicroRNAs
UTR	Untranslated region
lncRNAs	Long non-coding RNAs
SP20H	SUPT20H
NK cells	Natural killer cells
DCs	Dendritic cells
TAMs	Tumor-associated macrophages
EMT	Epithelial–mesenchymal transition
MMPs	Matrix metalloproteinases
ROS	Reactive oxygen species
TM4SF1	Transmembrane 4 superfamily member 1
MVP	Major vault protein
CSCs	Cancer stem cells
ECM	Extracellular matrix
M2-TAMS	M2-type tumor-associated macrophages
MDSCs	Myeloid-derived suppressor cells
Treg cells	Regulatory T cells
TANs	Tumor-associated neutrophils
TAFs	Tumor-associated fibroblasts
M1-TAMS	M1-type tumor-associated macrophages
sB7-H3	Soluble B7-H3
circRNAs	Circular RNAs
HER2	Human epidermal growth factor receptor 2
FR	Folate receptor
IL-11Rα	Interleukin-11 receptor α-chain
ACT	Adoptive cell therapy
CARs	Chimeric antigen receptors
scFv	Single-chain variable fragment
PDX	Patient-derived xenograft
CDX	Cell line-derived xenograft
PBD	Pyrrolobenzodiazepine
PDT	Photodynamic therapy
CDT	Chemodynamic therapy
PTT	Photothermal therapy
PARP	Poly ADP-ribose polymerase
